# The Expression of Transcription Factors is Different in Papillary Thyroid Cancer Cells during TNF - α induced EMT

**DOI:** 10.7150/jca.53349

**Published:** 2021-03-10

**Authors:** Nannan Lv, Fei Liu, Lan Cheng, Feng Liu, Jinsong Kuang

**Affiliations:** Department of Endocrinology and Metabolism, The Fourth People's Hospital of Shenyang; 20 Huanghe South st, Huanggu District, Shenyang,China.

**Keywords:** EMT, NF-κB, PTC, TNF-α, transcription factors.

## Abstract

Proinflammatory factor tumor necrosis factor-α (TNF-α) is an important inflammatory mediators in tumor microenvironment and autoimmune diseases, it is highly expressed in many solid tumors and tumor microenvironment, showing a tumor promoting role. However, the molecular mechanisms underlying TNF-α-increased invasion of thyroid cancer are still not fully understood. In order to explore whether TNF-α plays a key role in the process of epithelial mesenchymal transition (EMT) in papillary thyroid carcinoma (PTC), we used TNF-α to induce EMT in different PTC cell lines, and observed the expression of different transcription factors and signal pathways.

After TNF-α treatment, in TPC-1, Snail and ZEB2 mRNA levels did not change significantly, while Slug, Twist1, ZEB1 mRNA expression increased. In BCPAP, Snail mRNA level increased significantly (P < 0.01), while Twist1 showed a certain degree of increase only at the concentration of TNF - α 20 ng / ml (P < 0.01), but mRNA of Slug, ZEB1, ZEB2 showed no significant change. The expression of proteins was consistent with genes. The activation of different pathways did not show gene differences, and pathway inhibitors could reduce the invasion and metastasis of cells, but only NF-κB inhibitors could reverse the expression of transcription factors.

Expressions of Snail and Slug in different PTC cell lines were dependent on pro-oncogene mutation, but the pathway had no differences. The establishment of this study model can enrich the research on the pathogenesis and metastasis of thyroid cancer, effectively link the inflammatory microenvironment with the occurrence and development of thyroid cancer.

## Introduction

The tumor necrosis factor-α (TNF-α) is a cytokine released in the process of chronic inflammation and immune response, which plays an important role in activation of inflammation and immune cells, cellular homeostasis, and tumor progression 1, 2. It was originally named for its anti-tumor effect, but in recent years, more and more evidence show that TNF-α is an important mediator of inflammatory related cancers, and play a role as a tumor-promoting factor 1, 2. Although we found that TNF-α contributes to oncogenicity, but whether it play a role in pro-cancer or anti-cancer depending on the balance of promoting tumor and inhibiting tumor cytokines, as well as their relative concentrations, the expression state of their receptors, and the activation of surrounding cells 2, 3.

EMT is an important cell remodeling process occurred in embryogenesis, inflammation, wound healing and cancer. In EMT, epithelial cells phenotype changed, obtained mesenchymal cells characteristic. The interactive structure of epithelial cells such as tight junctions, adherens junctions, desmosomes and gap connection were lost, then the cellular actin cytoskeleton restructured, the protein expression profile changed in this process 4, 5. The decrease of E-cadherin is one of the landmark event in EMT. Obtaining mesenchymal phenotype in the process of EMT can increase cell mobile ability. For cancer cells, obtaining fibrous characteristics, in turn, can increase their activity to pass through the basement membrane of blood vessels or the lymphatic vessels 6. This is closely related to the tumor invasion and metastasis, and EMT became the first step in tumor metastasis, and key step. TNF-α is one of the inflammatory mediators which could promote EMT 1.

The global incidence of thyroid cancer increased rapidly, papillary thyroid cancer (PTC) accounted for 85%. According to the American statistics, in 1994 to 2013, the overall incidence of thyroid cancer increased 3% annually, with increases in the incidence rate and thyroid cancer mortality rate for advanced-stage papillary thyroid cancer 7. Data from our country suggested that the incidence and mortality of thyroid cancer was on the rise during 2003-2007, rising at the rate of 14.51% and 1.42% each year respectively 8. Papillary thyroid cancer is usually well differentiated, but the patients with distant metastasis, the 5-year survival rate is only 40% 9. Major effort has been made to understand the mechanisms behind the invasion and metastasis of thyroid carcinoma. Part of effort is devoted to cytokines and their molecular alterations in the malignant progression. Studies have shown that inflammatory cytokines TGF-β and EGF can induce EMT in thyroid cancer and thyroid cells cultured *in vitro* 10, 11; through the SMAD, NF-κB, AKT/GSK-3β, JAK/STAT signaling pathway, long-term low dose of TNF-α and IFN-γ stimulation can also induce invasion and metastasis in other tumors 12-15.

However, to date, we know little about the role of TNF-α in PTC. In order to explore whether TNF-α play a key role in invasion and metastasis of PTC and EMT, and further explore the molecular mechanism of which may involve in, we used TNF-α induced EMT in PTC cell lines, observed expression of different transcription factors and signaling pathway activation. Moreover, we examined the change of transcription factors expression and tumor mobile ability after given the signal pathway inhibitors.

## Materials and Methods

### Cell lines

Human papillary thyroid cancer cell lines used in this study were TPC-1 (RET mutation), BCPAP (BRAF mutation), K1 (BRAF mutation). TPC-1 was acquired from Dr. Haugen, Division of Endocrinology, Diabetes and Metabolism, University of Colorado Denver (Aurora, CO). BCPAP was purchased from The DSMZ-Deutsche Sammlung vonMikroorganismen und Zellkulturen GmbH (German Collection of Microorganisms and Cell Cultures; Braunschweig, Germany). K1 was purchased from The Health Protection Agency Culture Collections (Salisbury, United Kingdom). TPC-1 cells were cultured in high glucose DMEM, BCPAP and K1 were maintained in RPMI-1640 supplemented with 2 mmol/L L-glutamine (Gibco). All culture media were supplemented with 10% fetal bovine serum (FBS; Gibco). Cells were cultured at 37 °C in a humidified chamber supplemented with 5% CO2.

### Western blot assay

Cells were washed twice with ice-cold PBS and solubilized in RIPA buffer (Sigma-Aldrich, Saint Louis, MO) on ice and then was quantified using QuantiPro bicinchoninic acid assay kit (Sigma-Aldrich). Proteins were denatured at 100°C with sample buffer for 5 min. Equal amounts of protein (50 μg) separated by electrophoresis in 10% to 12% SDS-PAGE gels according to their molecular weight. Proteins were transferred onto PVDF membranes (Bio-Rad) and blocked for 2 h in blocking solution (5% nonfat dry milk in TBS containing 0.1% Tween 20). The membrane was then exposed to the primary antibody overnight at 4°C. Snail, slug, NF-κB, p-NFκB P65 (Ser536), STAT3, p-STAT3, AKT, p-AKT were purchased from Cell Signaling (Beverly, MA); Twist1, Zeb1, Zeb2, vimentin, E-cadherin, N-cadherin, β-action were purchased from Santa-Cruz Biotechnology, all primary antibodies dilution is 1:1000. After washing, the membranes were incubated for 1.5 h at room temperature with peroxidase-linked secondary antibody (Santa-Cruz). Signals were revealed with an electrochemoluminescence (ECL) Western Blotting Analysis System (Pierce) using the FluorChem®FC2 (Alpha Innotech, CA), and then quantified using ImageJ® software.

### Immunofluorescence

Coverslip covered with cells seeded in six-well plates, TNF-α treated 36 hours, when the fused cells grew to 95% -100%, was removed from the incubator, the cell culture medium was discarded. Cells were fixed with 4% paraformaldehyde for 20 min on the ice, 0. 1% of Triton X - 100 at room temperature for 5 min, and blocked with 1% BSA for 30min. Then samples were incubated with primary antibodies E-cadherin (1:200), N-cadherin (1:200), vimentin (1:50) which were used as described in western blot at 4℃ over night, and were incubated avoid light with Alexa secondary antibodies (Invitrogen, 1:400) at room temperature for 1 h. After washing, wells were covered with DAPI (Invitrogen). Preserved void light at 4℃ for microscopic examination.

### RNA extraction and qRT-PCR

Total RNAs were extracted using TRIzol reagent (Invitrogen, Life Tech-nologies, Grand Island, NY, USA) and reverse transcribed using PrimeScript^TM^ RT reagent Kit (TaKaRa). Resulting cDNAs were analyzed in triplicates using SYBR^®^* Premix Ex Taq*^TM^ (TaKaRa). Relative mRNA concentrations were determined by 2-(Ct-Cc) where Ct and Cc are the mean threshold cycle differences after normalizing to GAPDH values. For monitoring mRNA expression, primer sequences for RT-PCR were: SNAI1 (SNAIL) forward: 5′-CCTCCCTGTCAGATGAGGAC- 3′, SNAI1 (SNAIL) reverse: 5′-CCAGGCTGAGGTATTCCTTG-3′; SNAI2 (SLUG) forward: 5′-TTCGGACCCACACATTACCT-3′, SNAI2 (SLUG) reverse: 5′-GCAGTGAGGG CAAGAAAAAG-3′; TWIST1 forward: 5′-GGAGTCCGCAGTCTTACGAG-3′, TWIST1 reverse: 5′-TCTGGAGGACCTGGTAGAGG-3′; ZEB1 forward: 5′-GATGATGAATGCGAGTCAGATGC-3′, ZEB1 reverse: 5′-ACAGCAGTGTCTTGTTGTTGTAG-3′; ZEB2 forward: 5′-AACAACGAGATTCTACAAGCCTC-3′, ZEB2 reverse: 5′-TCGCGTTCCTCCAGTTTTCTT-3′; GAPDH forward: 5′-ACCCAGAAGACTGTGGATGG-3′, GAPDH reverse: 5′-TCTAGACGGCAGGTCAGGTC-3′.

### Wound healing assay

Cells were treated with TNF-α for 36 hours, then seeded into 12-well plates after digestion, cells number covered with the plate bottom was appropriate. A scratch was made through the center of each well using a 10-µl pipette tip, ensured the consistency of each scratch width. Plates were washed three times with PBS, and wash away cellular debris generated from scratches. Cells were cultured with serum- free medium, recorded by photographic. The plates were placed in incubator, photographs taken every 4-6 hours. Analysis of experimental results based on data collected pictures.

### Cell migration and invasion assays

Coated surface of the upper chamber of costar transwell chambers (8-μm pore size) with 50mg/L of Matrigel gel (1: 7 dilution), dried at 4 ℃. Then removed the residual liquid, hydrated basement with 50 μL serum-free medium containing 10g/L BSA at 37 ℃ for 30min. Treated cells with TNF-α (Invitrogen) and different pathway inhibitors ( all from Sigma) for 36 hours before preparing cell suspension, the cells were resuspended with serum-free medium containing BSA after digesting, adjusting the density to 5 × 10^5^. 200 μL cell suspension were seeded in the top chamber, 500 μL normal culture medium were added to the lower chamber. Cultured cells 36 hours, then gently wipe off Matrigel gels and indoor cells with a damp cotton swab, carefully removed the upper chamber, with a line tied down, and well marked. Fixed in 4% formaldehyde and stained with hematoxylin and eosin, randomly selected six counts horizons of cells attached to the under surface of the membrane at high magnification (× 400). The experiment was repeated three times.

### Statistical analysis

Count data presented as the mean and standard deviation (mean + SEM), was processed by SPSS 20 statistical software. Compared with the average with One-ways ANOVA LSD analysis, homogeneity of variance with Dunnett 's T3, P < 0.05 was considered statistically significant difference.

## Results

### Effects of TNF-α treatment on morphology and epithelial mesenchymal transition related markers in papillary thyroid carcinoma cell lines

**Figure 1:**
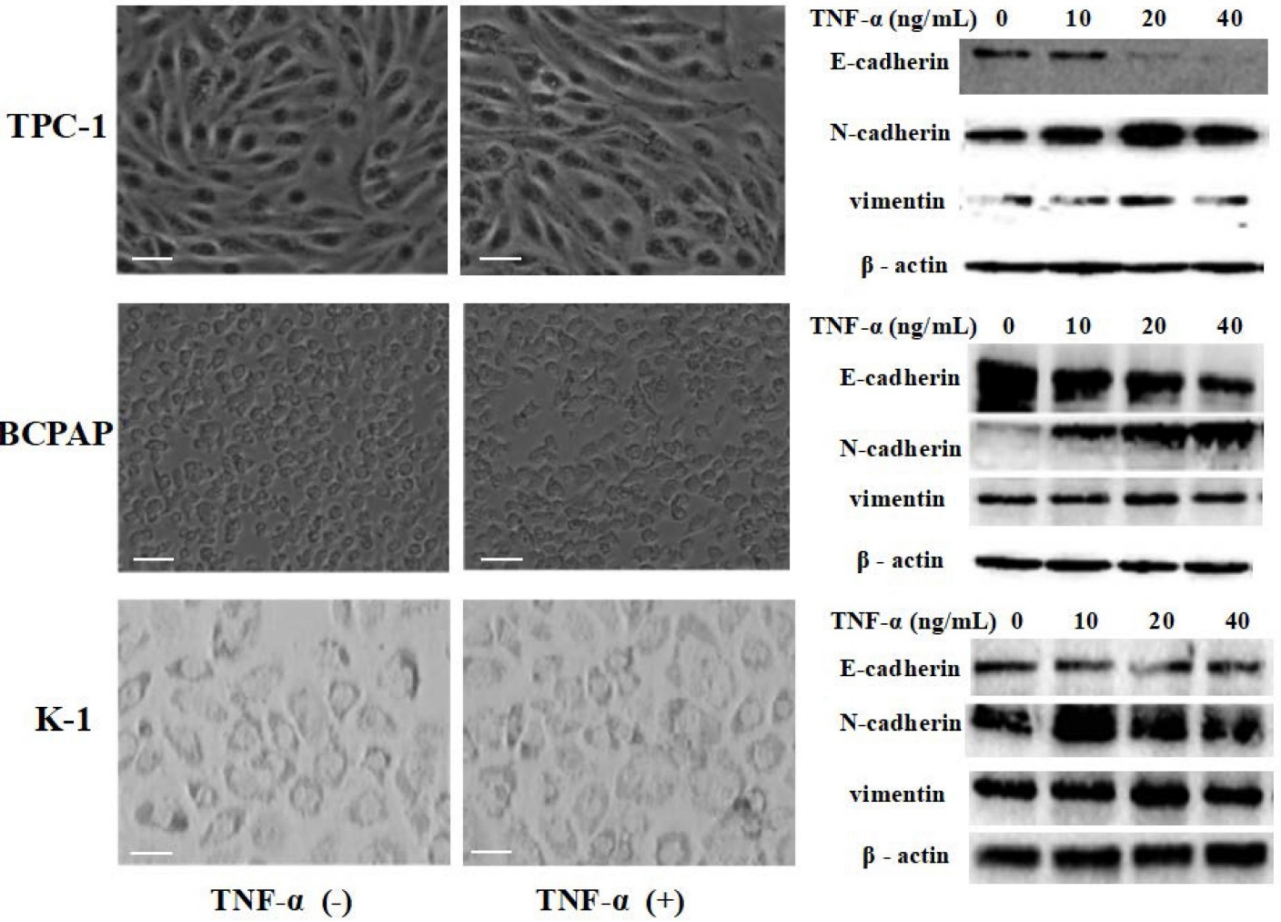
** TNF-α exposure induced transition of the epithelial to the mesenchymal-like phenotype in cultured Papillary thyroid cancer cells TPC-1, BCPAP, K1.** Three independent experiments were done. Cancer cells were serum-starved for 24 h before treatments. Cells were treated with medium only medium, medium with 20 ng/mL TNF-α, and morphology was examined and photographed using a phase-contrast microscope. At 36 h unstimulated cells retained their epithelial phenotype. In contrast, cells treated TNF-α displayed detached mesenchymal-like morphology. EMT markers E-cadherin, N-cadherin and vimentin were detected. Total protein was isolated and subjected to Western blot, expression of β-action serves as a loading control. Magnification, × 400, scale bars: 20 µm.

Papillary thyroid carcinoma cell lines TPC-1 (identified with RET mutation), BCPAP (identified with RET mutation) and K1 (identified with RET mutation) were exposed to different concentrations of TNF-α (0, 10 ng/mL, 20 ng/mL, 40 ng/mL) for 36 h, then extracted protein and mensurated the changes of classical epithelial mesenchymal transition (EMT) markers: E-cadherin, N-cadherin and vimentin. As shown in Fig.1, Western blot results revealed that the level of E-cadherin protein in TPC-1 cells was decreased, N-cadherin and vimentin protein were increased. However, the increase rate of vimentin was not very clear, showing high expression only at 20 ng/mL concentration. The situation in BCPAP and K1 was similar to TPC - 1. Further more, we observed cell morphology changes under the influence of TNF-α (20 ng/mL), results were shown in Fig. 1. TPC-1 cell morphology gradually changed from the original fat round to spindle-shaped, which form can make the cells more likely to move. This phenomenon was also reflected in BCPAP, but it was not obvious in K1. K1 cells did not change to a slender spindle-shaped, but showed pleomorphic changes. The results of this part together suggested that TNF-α successfully induced epithelial mesenchymal transition in papillary thyroid carcinoma cells, and optimal results of various marker protein level could be obtained under the concentration of 20 ng/mL. Therefore, the following research we used this concentration.

### Localization of epithelial mesenchymal transition markers after TNF-α treatment

**Figure 2:**
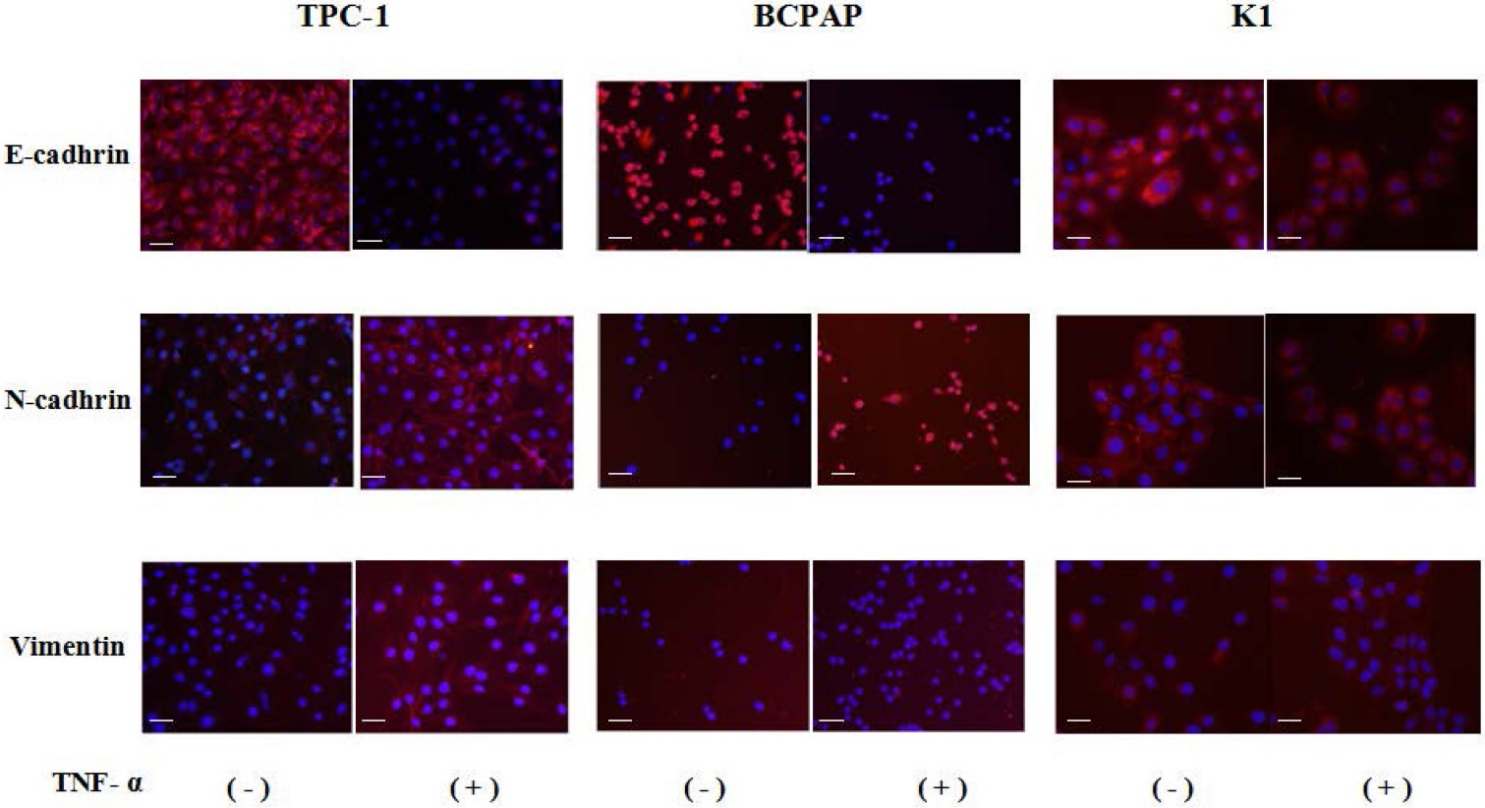
** Immunofluorescence images of EMT markers in human Papillary thyroid carcinoma cells TPC-1, BCPAP and K1.** The three kinds of cells were treated with TNF-α (20 ng/mL) for 36h, respectively. Expression of E-cadherin (red), N-cadherin (red) and vimentin (red) were analyzed by immunofluorescence staining. Nuclei were visualized with DAPI staining (blue). Magnification, × 200, scale bars: 20 μm.

Cell immunofluorescence results showed that after co-cultured with the optimal concentration of 20 ng/mL of TNF-α for 36 hours, in the three kinds of cell lines, N - cadherin was mainly located in cell membranes, and E-cadherin and vimentin were mainly located in cytoplasm, and presented a decreases of E-cadherin, N-cadherin and vimentin expression levels increased, as shown in Fig. 2, the above results consistent with the results of Western.

### Expression of transcription factors SNAI1, SNAI2, TWIST1, ZEB1, ZEB2 in human PTC cells exposure to TNF-α

**Figure 3:**
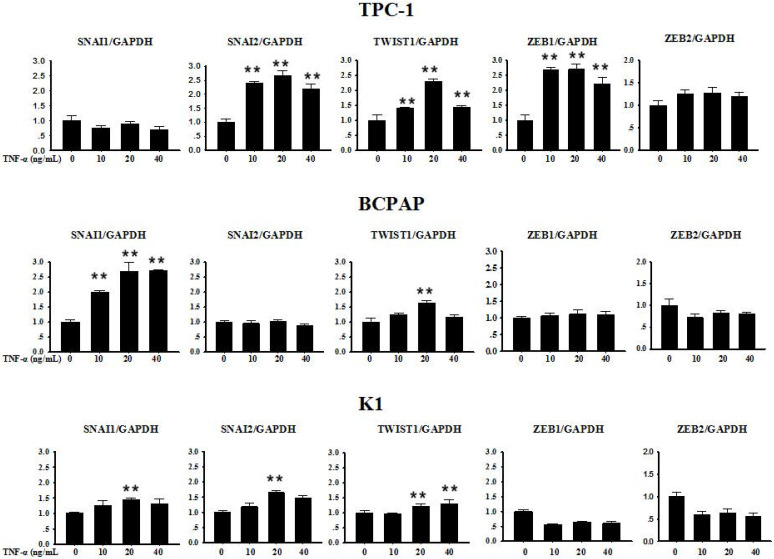
** Expression of SNAI1, SNAI2, TWIST1, ZEB1 and ZEB2 mRNA in Papillary thyroid carcinoma cells TPC-1, BAPAP and K1 by qRT-PCR.** Cancer cells were serum-starved for 24 h before treatments then cells were treated with different concentrations of TNF-α as provided for 12 h. Total RNA was isolated and subjected to qRT-PCR, values were normalized with GAPDH used as an internal control.Three independent experiments were done. Columns, mean (n = 3); bars, SEM. **P < 0.01

Several transcription factors are seen as upstream coordinators of the complex events that together make up EMT. Often the expression of Snail family genes Snail, Slug and other E-cadherin repressors such as Zeb1, Zeb2 and Twist1 can be detected at sites of EMT in the leading edge of an invading tumour by immunohistochemically technique 5. In order to explore whether the above described transcription factors were involved in TNF-α-induced EMT in PTC cell lines, we first collected cells precipitation to extract RNA after TNF-α acted on cells for 12 h, and mRNA levels transcription factors were measured. As shown in Fig. 3, in TPC-1, SNAI1 (Snail) and ZEB2 mRNA levels did not change significantly, while the mRNA expression of SNAI2 (Slug), TWIST1, ZEB1 were increased in different degree compared with the control group, the differences were statistically significant. In BCPAP, the level of SNAI1 increased obviously (*P<0.01*), while TWIST1 showed a certain degree of increase only under the condition of TNF-α 20 ng/mL (*P<0.01*), but levels of SNAI2, ZEB1, ZEB2 showed no obvious change. The situation in K1 was similar to BCPAP, SNAI1, SNAI2, TWIST1 showed weak increase (*P<0.01*), and ZEB1, ZEB2 had no significant difference before and after drug treatment.

### Protein status of transcription factors and signaling pathway responded to TNF-α

**Figure 4:**
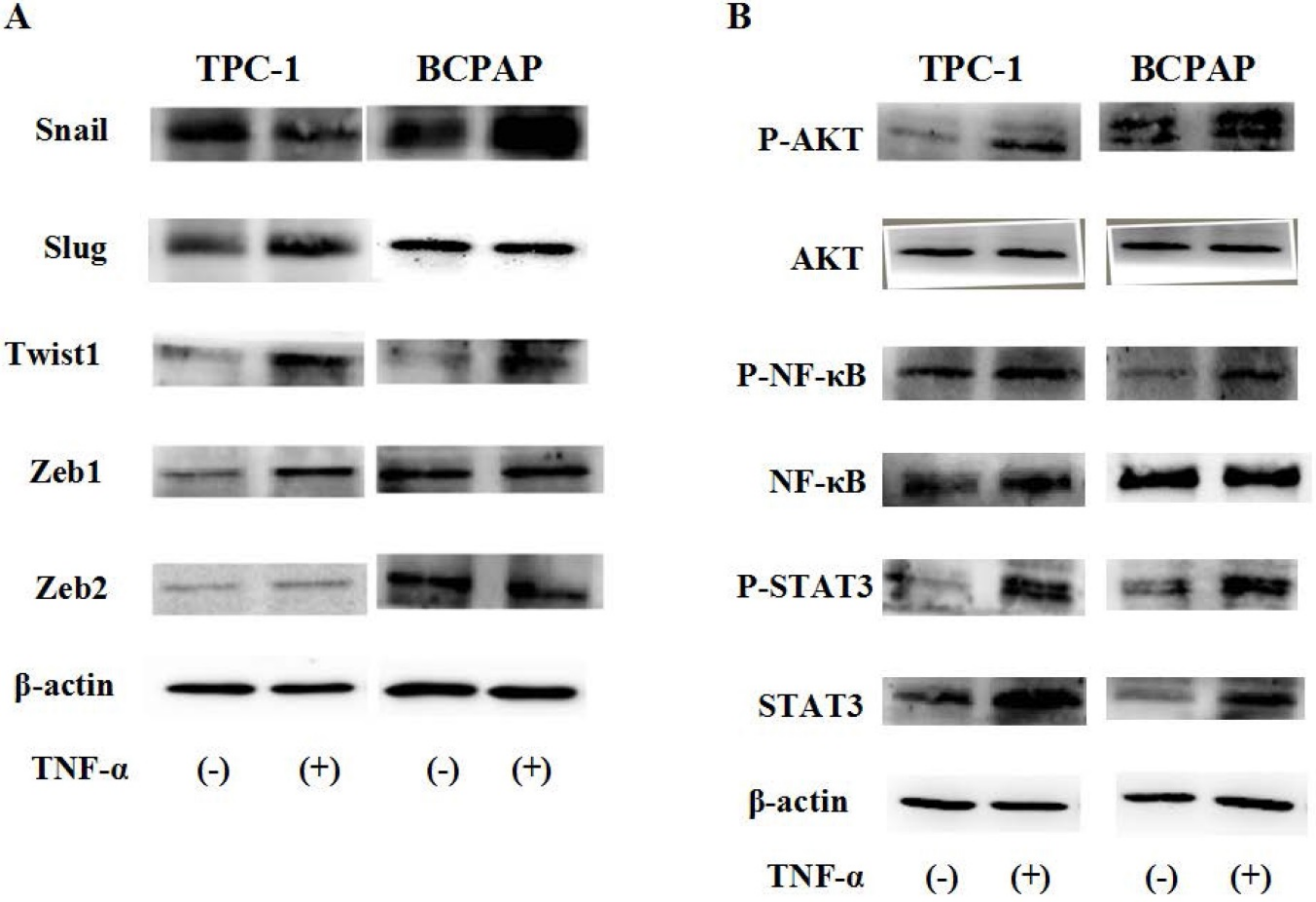
** Protein status of transcription factors and signaling pathway responded to TNF-α.** Human Papillary thyroid carcinoma cells TPC-1, BCPAP were used in these studies. Cancer cells were serum-starved for 24 h before treatments. (A)Transcription factors Snail, Slug, Twist1, Zeb1, Zeb2 protein levels were detected. (B) TNF-a activated Akt, NF-kB and STAT3 signaling pathways. Total cell lysates were extracted and subjected to Western blot analyses, expression of β-action serves as a loading control. The experiment was performed in triplicate.

Due to post transcriptional regulation is very important to determine the structure and function of cell protein, so just mRAN result was not enough, then we used Western blot to further verify the protein level changes of these transcription factors in cell TPC-1 and BCPAP. As shown in Fig. 4A, the results showed that in TPC-1, responded to TNF**-**α (20 ng/mL) for 36 hours, Snail was down regulated, Slug, Twist1, Zeb1 were up-regulated, but there was no significant change in Zeb2 at the protein levels. In BCPAP, protein expression levels of Snail and Twist1 was increased, Slug, Zeb1 and Zeb2 were unchanged. From the results given above we speculated that transcription factors Snail and Slug responded to TNF-α may depend on the gene mutation in different cell lines.

To explore what caused such difference, was it determined by the function caused by different signaling pathways depend on the nature of the cells themselves, we examined the changes of ATK, NF-κB, STAT3 signaling pathways induced by TNF**-**α (20 ng/mL for 2h). The results in Fig. 4B reflected that above signaling pathways all were activated by TNF-α, showed no differences in the TPC-1 and BCPAP cell lines.

### Effect of inhibitors on cell mobile ability and variety of transcription factors mediated by TNF-α

**Figure 5:**
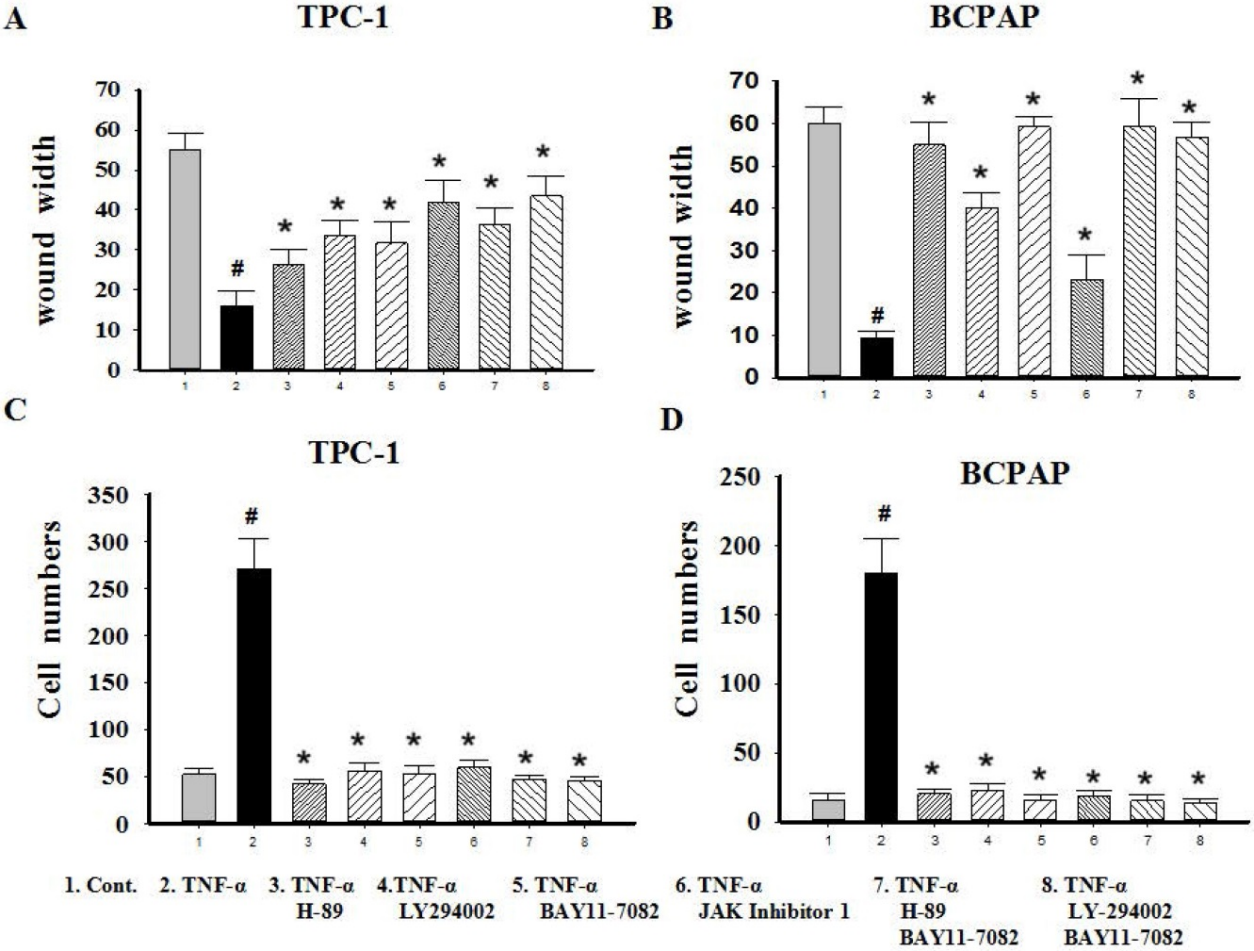
** Migration and invasion capacity changes after signaling pathway inhibitors treatment in Papillary thyroid carcinoma cells TPC-1 and BAPAP.** (A) TPC-1 and (B) BCPAP migration ability were detected using wound healing assays after pretreatment with H-89 (50 μM), LY294002 (20 μM), BAY11-7082 (50 nM), JAK inhibitor 1 (5 μM) for 30 min and followed by stimulation wih TNF-α (20 ng/mL) for 36 h. (C) TPC-1 and (D) BCPAP invasion capacity were detected using transwell assays after treatment indicated above. The results are representative of at least three independent experiments, Error bars represent S.E.M. *P < 0.05

**Figure 6:**
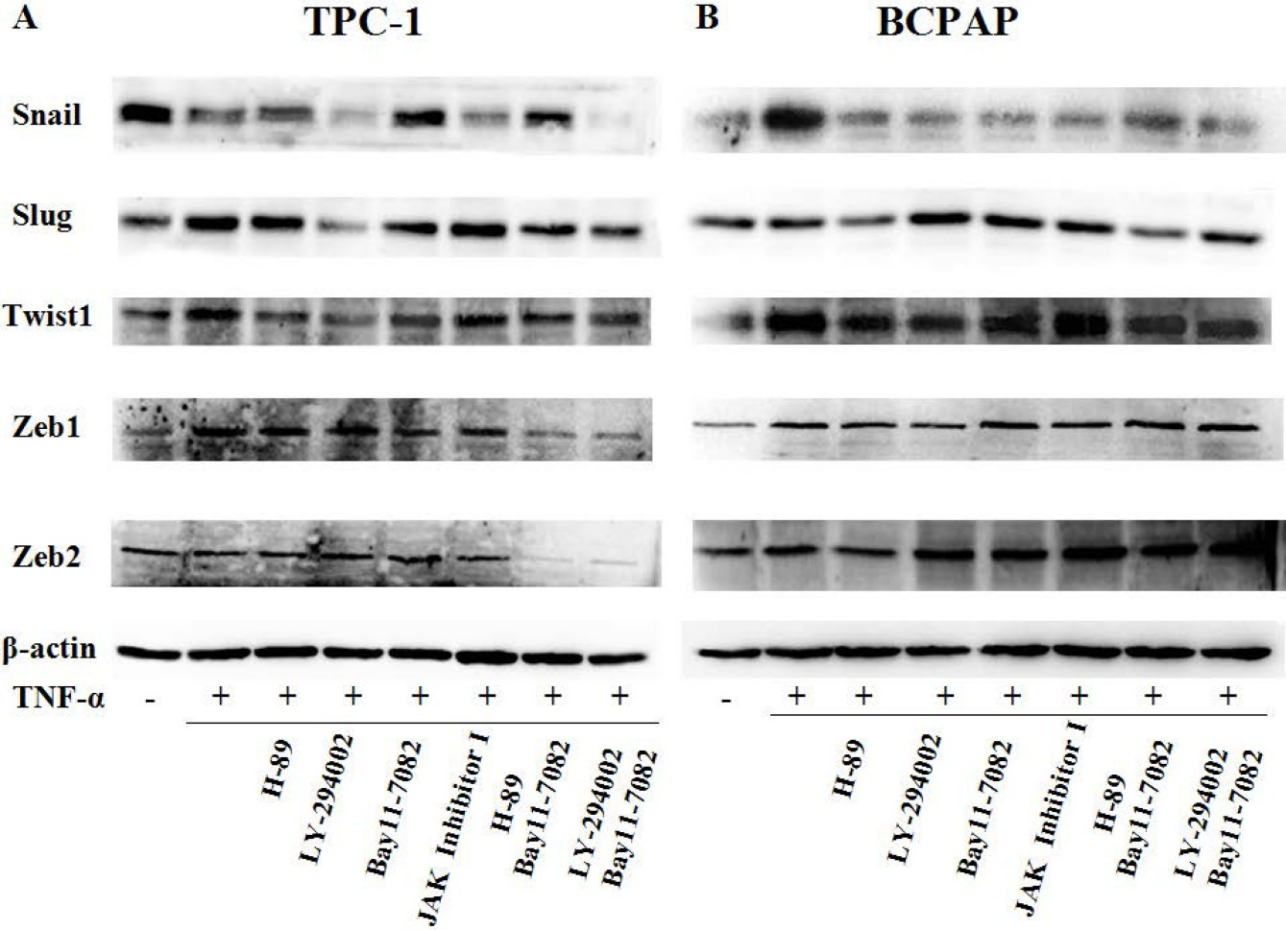
** TNF-α regulates expression of transcription factors through NF-κB pathway.** (A) TPC-1 and (B) BCPAP were pretreated with H-89 (50 μM), LY294002 (20 μM), BAY11-7082 (50 nM), JAK inhibitor 1 (5 μM) for 30 min and followed by stimulation wih TNF-α (20 ng/mL) for 36 h. The expression of Snail, Slug, Twist1, Zeb1, Zeb2 were examined by western blotting, expression of β-action serves as a loading contro.The results are representative of at least three independent experiments.

Since TNF-α can induce the activation of these pathways (showed in Fig. 4B), and ATK can be activated by cAMP/PKA and/or PI3K, inhibitors of PKA (H89), PI3K/AKT (LY294002), NF-κB (BAY11-7082), JAK/STAT3 (JAK inhibitor 1) were used to investigate the molecular mechanisms underlying TNF**-**α mediated the variety of transcription factors in PTC cells. TPC-1 and BCPAP cells were pretreated with inhibitors for 30 min before TNF**-**α stimulation, 36 h later, the cell invasion and metastasis ability was detected by wound healing and transwell assays, the expression of these transcription factors were determined by western blot. The results of wound healing assay showed that after 24 hours of climbing, the migration ability of TPC-1 and BAPAP with drug treatment group were improved, after adding inhibitors, cell migration ability were descended (Fig. 5A and 5B). Similarly, in the transwell assays, TNF-α could significantly enhance the cells invasion ability, but after given the inhibitors, cell invasion ability were restricted (Fig. 5C and 5D). Although these inhibitors could make TNF-α induced cell invasion and metastasis decline, but only NF-κB inhibition was able to reverse the expression of Snail, Slug, Twist1, Zeb1 in TPC-1 and Snail, Slug, Twist1 in BCPAP (Fig. 6). The above results indicated that TNF-α mediates variety of transcription factors via activation of NF-κB signaling pathway, not others.

## Discussion

In this study, we investigated the influence of pro-inflammatory cytokines TNF-α on EMT in PTC cells *in vitro*. We found the landmark change of EMT was initiated in PTC cells exposure to TNF-α: the levels of E-cadherin expression were downregulated, the levels of N-cadherin and Vimentin expression were upregulated, and cell morphology changed (Fig. 1 and 2). Three kinds of PTC cells lines TPC-1, BCPAP and K1 (identified with RET mutations and BRAF mutation, RET gene rearrangements (RET/PTCs) represent together with BRAF pointmutations the two major groups of mutations involved in papillary thyroid carcinoma (PTC) initiation and progression 16), whose response to TNF-α were similar, suggesting that proto-oncogene mutation in PTC cells did not affect the changes of E-cadherin expression levels.

Epithelial mesenchymal transition (EMT) initially is often reported in the embryonic development, but now, more studies reported that it was closely associated with tumor metastasis. And, it also seems to be common in papillary thyroid carcinoma invasion and metastasis 17. The transfer involves not only the changes of cells, but also tumor microenvironment. Tumor cells and/or tumor associated immune cells and inflammatory cells can produce cytokines, and these cytokines may play a direct role in tumor metastasis 18. Studies have shown inflammatory cytokines TGF -β can induce EMT in human thyroid cancer cells *in vitro* 19. But whether TNF-α can induced EMT in papillary thyroid carcinoma and the possible mechanisms involved are still unknown. So we carry on with this research.

Many studies showed that transcription factors SNIA1/2, ZEB1/2, TWIST1 could initiate EMT by multiple pathways converging in the repression of epithelial-specific genes and activation of mesenchymal-specific genes. They also have been identified that were closely related to invasion and TNM stage in thyroid cancer 20- 22. So, in order to study mechanisms of TNF-α induced EMT in PTC, we observed the expression changes of above transcription factors.

First of all, we enter into the expression of Snail family members Snail and Slug. The major conclusions from our current study about them are: the expression changes of Snail and Slug by TNF-α occurred at the post-translational level and may be in a dynamic process. Slug plays a leading role in RET mutant PTC cell TPC-1, whereas Snail plays a leading role in BRAF mutant PTC cell BCPAP (Fig. 3 and Fig. 4). This is the first reported in thyroid carcinoma. Although studies have reported that increased expression of Snail was the markers changes in EMT and tumor invasion and metastasis, and knockout this gene can reverse EMT and tumor invasive ability 23. But there is also evidence revealed that the down-regulation of snail and up regulation of slug is interconnect with invasion and metastasis of tumor and poor prognosis 24, 25. The latter is consistent with our results obtained in TPC-1, we also found that the expression levels of Snail protein was reduced whereas Slug protein was increased under the action of TNF-α (Fig. 4A). Studies from Yasui et al and Baquero et al showed that the overexpression of snail can induce EMT and promote invasiveness in thyroid cancer cells with BRAF mutation 26, 27. This is also matched with the results we obtained in BCPAP (Fig. 4B). In a word, the mechanism of action of snail and slug in the cells is very complex, it is depending on the state of the cell and the pro-oncogene. So the further research on the mechanism by which they play a key role in the different mutations of thyroid carcinoma is still necessary. Our follow-up study showed that inhibiton of NF-κB in TPC-1 and BCPAP, the snail and Slug expression were all reversed, and the other pathway inhibitors did not present this effect (Fig. 6).

Secondly, in our study we found that TNF-α can induce the higher expression of Twist1 both in mRNA and protein levels, and this has nothing to do with the change of cell properties. Moreover, it was NF-κB not the other pathway played a key role in TNF-α -induced expression of Twist1 (Fig. 4 and Fig. 6). Although combination of TNF-α and its receptors can activate different signaling pathways, but the central position of the NF-κB has been widely recognized. As one of the upstream signals of Twist1, NF-κB regulating Twist1 is also mentioned in many studies. A study in 2012 pointed out that similar to the situation in drosophila, TNF-α in mammals is also to be activated the expression of Twist1 through the NF-κB signaling pathway. The study also found that higher TWIST1 expression could inhibit the NF-κB signaling pathway, in turn, inhibit TNF-α promoter. There is a negative feedback mechanism between the three, and thus they were maintained a dynamic balance 28. Pham et al's study pointed out that the activation of NF-κB was a necessary condition for up-regulated the expression of Twist1, Twist1 could be to act against programmed death caused by chemotherapeutic drugs or TNF-α in NF-κB deficient cells 29. A cancer researches results also proved that NF-κB mediated Twist1 expression was the key step in the TNF-α inducing EMT 30. The results of the study showed us the TNF-α/NF-κB/Twist1 signaling pathway was related to apoptosis, invasion, metastasis and EMT in cancer cells.

Thirdly, we found only Zeb1 showed the mRNA and protein levels upregulation in response to TNF-α, there was no significant change in Zeb1 expression in BCPAP. We did not observe any change in Zeb2 in neither two cells. Similarly, Zeb1 in TPC-1 was also regulated by the NF-κB pathway. Mato's study showed that high expression of ZEB1 were found in TPC-1 selected cells which expressing the ABCG2/BCRP gene, and the knockdown of ZEB1 promoted nuclear re-expression of E-cadherin, reduced expression of vimentin, N-cadherin. Analysis of human thyroid carcinoma showed ZEB1 genes showed higher expression at stages III and IV than at stages I and II 31. In addition, there is study reported that PGI/AMF over-expression could lead to increased DNA binding activity of NF-κB, which transcriptionally up-regulates the expression of ZEB1 and ZEB2, resulting in the induction of EMT 32. The results of these studies were similar to ours, suggesting that ZEB1 as one of EMT induced genes, play a key role in invasion and metastasis of TPC-1, and this regulation is depends on the NF-κB signaling pathways. Although some studies have shown ZEB2 can regulate tumor EMT, and a high ZEB2/E-cadherin ratio predicted poor overall survival 33,34; there are also some studies have shown expression of ZEB2 has nothing to do with EMT nor clinicopathological outcomes 35.

In addition, although the role of the pathway inhibitors in regulating the expression of transcription factors were not the same, but they all could decrease invasion and metastasis ability of cells exposure to TNF-α (P<0.05, Fig. 5). This suggests that cancer cells invasion and metastasis is a complex process, in addition to EMT, there may be other mechanisms involved. We should appreciate further clarification.

We demonstrated that expression of transcription factors SNAI1/2, TWIST1, ZEB1/2 in TNF-α-induced EMT were different. Of which TWIST1 showed an upregulation both in mRNA and protein levels, and there is no difference between the cells; while SNAI1/SNAI2 is showing a mechanism of post-transcriptional regulation after TNF-α stimulation, and behave differently in different cells: Slug plays a leading role in RET mutant PTC cell TPC-1, whereas Snail plays a leading role in BRAF mutant PTC cell BCPAP; ZEB1 mRAN and protein levels were increased only in the TPC-1; ZEB2 were not found obvious changes in the two kinds of cells. The regulation of transcription factors of the above is mediated by NF-κB, because after NF-κB was inhibited, the changes of these transcription factors presented reversals at the same time.

In summary, low concentration of TNF-α can induce EMT in different papillary thyroid cancer cell lines. The expression of Snail and Slug is related to the mutation of BRAF gene. In the cells with BRAF mutation, the expression of snail increases, while slug decreases; in the cells without BRAF mutation, the expression of snail decreases, while slug increases. These processes involve changes of many pathways, but only inhibition of NF-κB pathway can reverse the expression of these transcription factors. This is a novel study that has important implications to understanding the molecular mechanisms of thyroid tumorigenesis, particularly the invasiveness and metastasis of Papillary thyroid carcinomas with different gene mutation types. The establishment of this study model can enrich the research on the pathogenesis and metastasis of thyroid cancer, effectively link the inflammatory microenvironment with the occurrence and development of thyroid cancer, and provide certain theoretical basis for finding effective biological therapeutic targets.

## Supplementary Material

Supplementary figures and tables.

## Abbreviations

BRAF: B-type Rapidly Accelerated Fibrosarcoma kinase
EMT: epithelial mesenchymal transition
PTC: papillary thyroid cancer
mRNA: Messenger RNA
RT-PCR: Real Time- Polymerase Chain Reaction
TNF-α: Tumor Necrosis Factor-α
NF-κB: Nuclear Factor 'kappa-light-chain-enhancer' of activated B-cells
STAT3: Signal Transducers and Activators of Transcription 3
