# Supplementary material for: The Expression of Transcription Factors is Different in Papillary Thyroid Cancer Cells during TNF - α induced EMT
Source: J Cancer. 2021 Mar 10;12(9):2777–86. doi: 10.7150/jca.53349 (PMC8040707; doi:10.7150/jca.53349)

Fig.s1 Morphological change of induced cells

A **TPC-1**

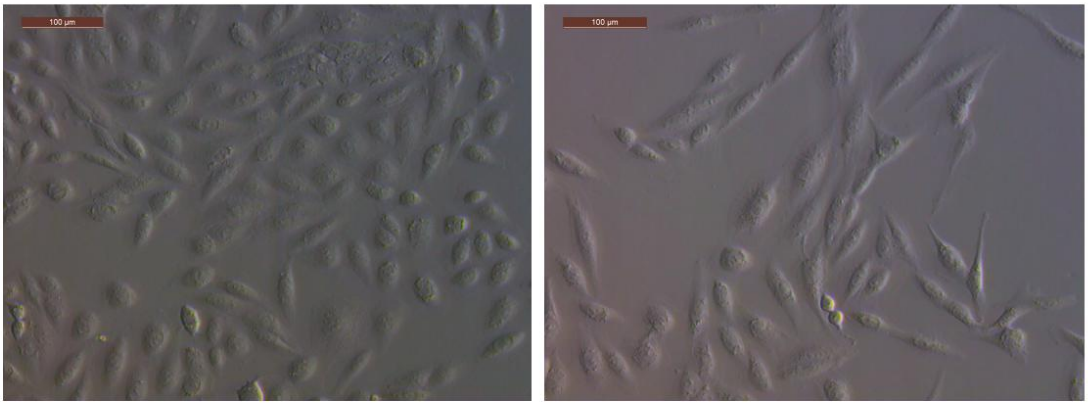

Cont. **TNF- α**

B **BCPAP**

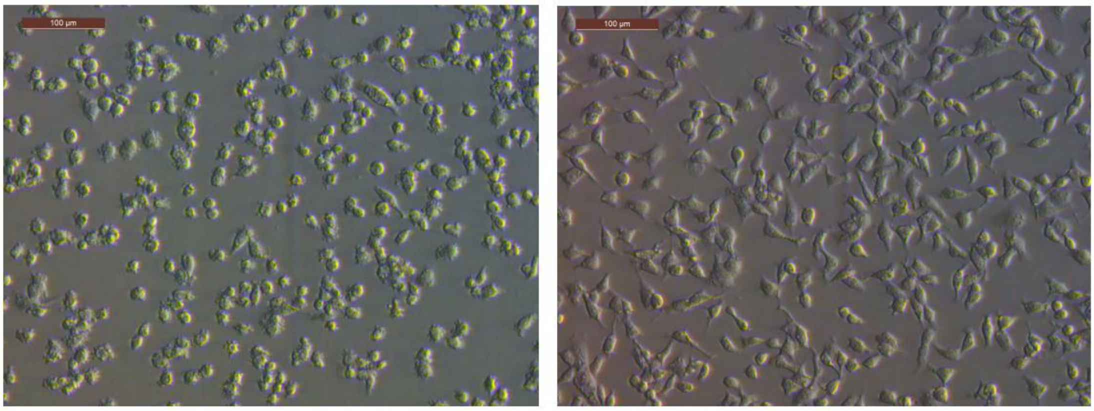

Cont. **TNF- α**

C **K1**

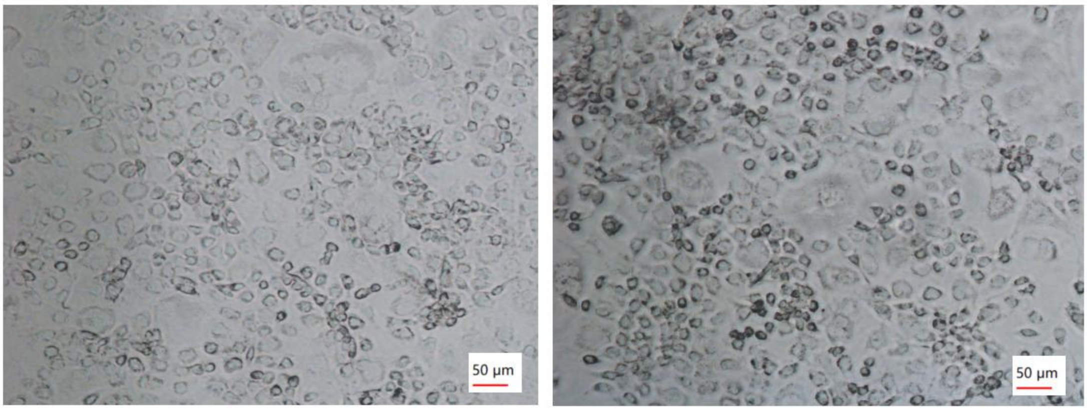

Cont. **TNF- α**

Fig.s2 **Immunofluorescence images of EMT markers in TPC-1**

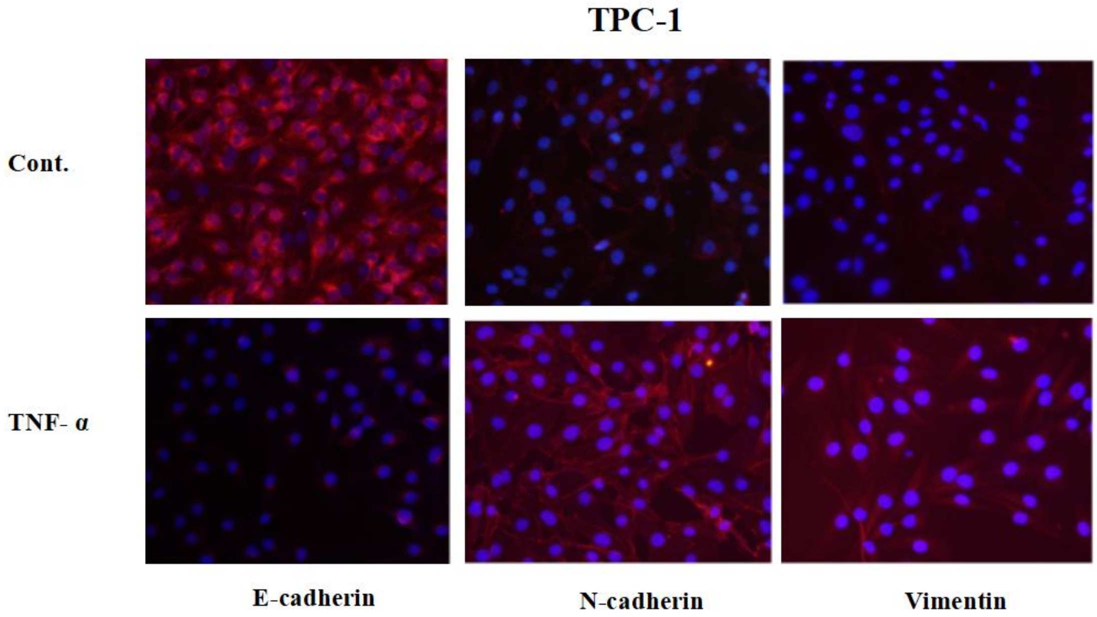

Fig.s3 **Immunofluorescence images of EMT markers in BCPAP**

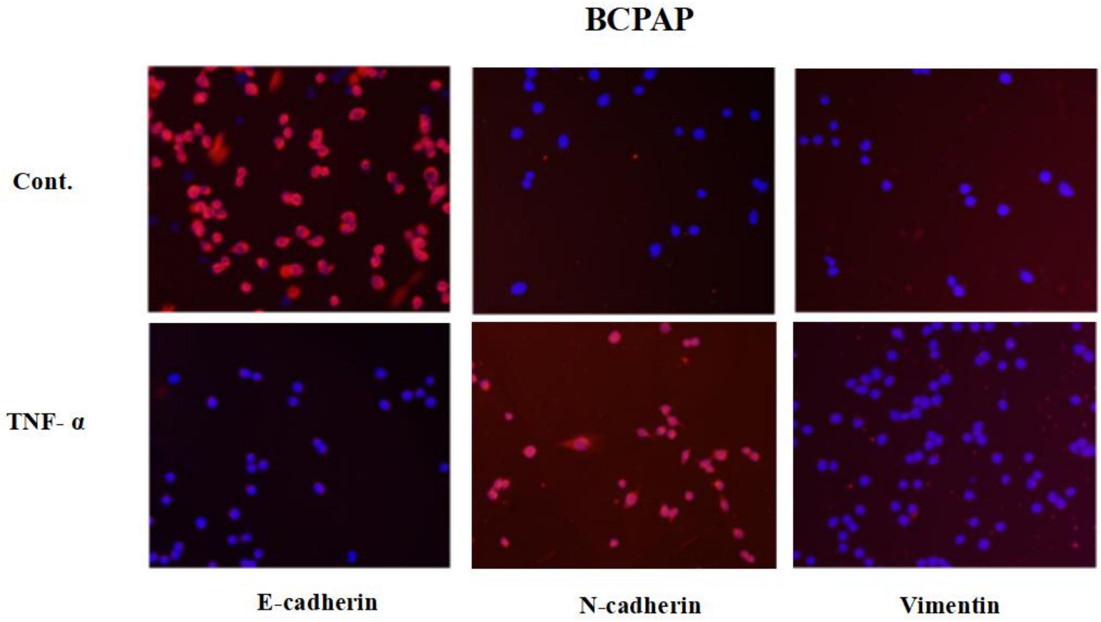

Fig.s4 **Immunofluorescence images of EMT markers in K1**

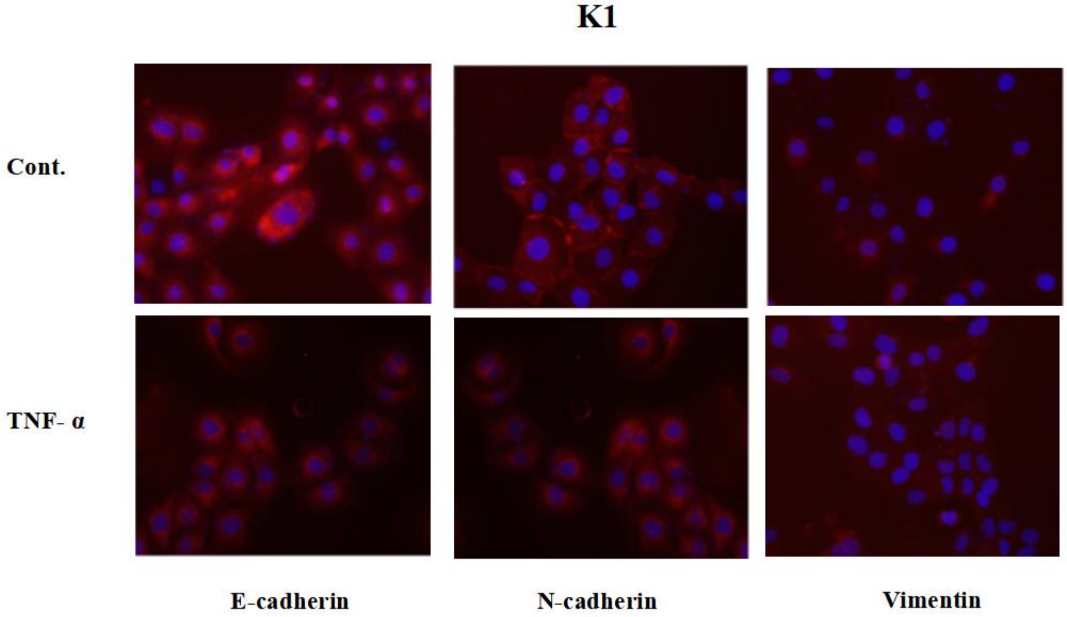

Supplement: Supplementary file 1 — Supplementary figures and tables. [file jcav12p2777s1.pdf]
